# Supplementary material for: Correlation between the Antibiotic Resistance Genes and Susceptibility to Antibiotics among the Carbapenem-Resistant Gram-Negative Pathogens
Source: Antibiotics (Basel). 2021 Mar 4;10(3):255. doi: 10.3390/antibiotics10030255 (PMC8001261; doi:10.3390/antibiotics10030255)
Supplement: Supplementary file 1 [file antibiotics-10-00255-s001.pdf]

Table S1. Antibiogram analysis results of the total Gram-negative isolates against different tested antimicrobial agents (n=194)

AA	*K. pneumoniae* (n=89)		*E. coli* (n=47)		*P. aeruginosa* (n=58)		Total (n=194)	
	S	R	S	R	S	R	S	R
	n° (%)	n° (%)	n° (%)	n° (%)	n° (%)	n° (%)	n° (%)	n° (%)
AX	13 (15)	76 (85)	11 (23)	36 (77)	16 (28)	42 (72)	40 (21)	154 (79)
AMC	41 (46)	48 (54)	35 (74)	12 (26)	21 (36)	37 (64)	97 (50)	97 (50)
CFR	14 (16)	75 (84)	14 (30)	33 (70)	14 (24)	44 (76)	42 (22)	152 (78)
CXM	16 (18)	73 (82)	11 (23)	36 (77)	16 (28)	42 (72)	43 (22)	151 (78)
CTX	16 (18)	73 (82)	12 (26)	35 (74)	32 (55)	26 (45)	60 (31)	134 (69)
FEP	47 (53)	42 (47)	23 (49)	24 (51)	41 (71)	17 (29)	111 (57)	83 (43)
MEM	67 (75)	22 (25)	47 (100)	0 (0)	46 (79)	12 (21)	160 (82)	34 (18)
ATM	36 (40)	53 (60)	29 (62)	18 (38)	52 (90)	6 (10)	117 (60)	77 (40)
DO	81 (91)	8 (9)	44 (94)	3 (6)	38 (66)	20 (34)	163 (84)	31 (16)
TE	40 (45)	49 (55)	19 (40)	28 (60)	34 (59)	24 (41)	93 (48)	101 (52)
CIP	44 (49)	45 (51)	20 (43)	27 (57)	34 (59)	24 (41)	98 (51)	96 (49)
LEV	53 (60)	36 (40)	28 (60)	19 (40)	36 (62)	22 (38)	117 (60)	77 (40)
AK	71 (80)	18 (20)	44 (94)	3 (6)	49 (84)	9 (16)	164 (85)	30 (15)
CN	56 (63)	33 (37)	42 (89)	5 (11)	47 (81)	11 (19)	145 (75)	49 (25)
TOB	45 (51)	44 (49)	38 (81)	9 (19)	37 (64)	21 (36)	120 (62)	74 (38)
SXT	41 (46)	48 (54)	25 (53)	22 (47)	19 (33)	39 (67)	85 (44)	109 (56)
AZM	55 (62)	34 (38)	39 (83)	8 (17)	nd	nd	94 (69)*	42 (31)*

* percentage relative to the total *K. pneumoniae* and *E. coli* isolates only

Abbreviations: AA: antimicrobial agent, S:sensitive, R:resistant, n°: number of isolates, %: approximate percentage, AX: amoxicillin, AMC: co-amoxiclav, CFR: cefadroxil, CXM: cefuroxime, CTX: cefotaxime, FEP: cefepime, MEM: meropenem, ATM: aztreonam, DO: doxycycline, TE: tetracycline, CIP: ciprofloxacin, LEV: levofloxacin, AK: amikacin, CN: gentamicin, TOB: tobramycin, SXT: co-trimoxazole, AZM: azithromycin, nd: not determined (due to lack of interpretation data in CLSI informational supplement).

Table S2. Antibiogram analysis results of the CRGNP

AA	*K. pneumoniae* (n=22)		*P. aeruginosa* (n=12)	
	Sensitive nº (%)	Resistant nº (%)	Sensitive nº (%)	Resistant nº (%)
AX	0 (0)	22 (100)	0 (0)	12 (100)
AMC	0 (0)	22 (100)	0 (0)	12 (100)
CFR	0 (0)	22 (100)	0 (0)	12 (100)
CXM	0 (0)	22 (100)	0 (0)	12 (100)
CTX	0 (0)	22 (100)	0 (0)	12 (100)
FEP	5 (23)	17 (77)	0 (0)	12 (100)
MEM	0 (0)	22 (100)	0 (0)	12 (100)
ATM	3 (14)	19 (86)	7 (58)	5 (42)
DO	21 (96)	1 (4)	5 (42)	7 (58)
TE	12 (55)	10 (45)	4 (33)	8 (67)
CIP	4 (18)	18 (82)	0 (0)	12 (100)
LEV	8 (36)	14 (64)	0 (0)	12 (100)
AK	11 (50)	11 (50)	4 (33)	8 (67)
CN	10 (45)	12 (55)	4 (33)	8 (67)
TOB	3 (14)	19 (86)	1 (8)	11 (92)
SXT	7 (32)	15 (68)	1 (8)	11 (92)
AZM	6 (27)	16 (73)	nd	nd

Abbreviations: AA: antimicrobial agent, nº: number of isolates, %: approximate percentage, AX: amoxicillin, AMC: co-amoxiclav, CFR: cefadroxil, CXM: cefuroxime, CTX: cefotaxime, FEP: cefepime, MEM: meropenem, ATM: aztreonam, DO: doxycycline, TE: tetracycline, CIP: ciprofloxacin, LEV: levofloxacin, AK: amikacin, CN: gentamicin, TOB: tobramycin, SXT: co-trimoxazole, AZM: azithromycin, nd: not determined (due to lack of interpretation data in CLSI informational supplement).

Table S3. Carbapenem-resistant *K. pneumoniae* isolates

AA	K1	K59	K6	K13	K33	K37	K43	K60	K61	K62	K64	K76	K78	K80	K81	K83	K44	K75	K77	K82	K88	K94
**AX**	R	R	R	R	R	R	R	R	R	R	R	R	R	R	R	R	R	R	R	R	R	R
**AMC**	R	R	R	R	R	R	R	R	R	R	R	R	R	R	R	R	R	R	R	R	R	R
**CFR**	R	R	R	R	R	R	R	R	R	R	R	R	R	R	R	R	R	R	R	R	R	R
**CXM**	R	R	R	R	R	R	R	R	R	R	R	R	R	R	R	R	R	R	R	R	R	R
**CTX**	R	R	R	R	R	R	R	R	R	R	R	R	R	R	R	R	R	R	R	R	R	R
**FEP**	R	R	S	R	S	S	R	R	S	R	R	R	R	R	R	R	S	R	R	R	R	R
**MEM**	R	R	R	R	R	R	R	R	R	R	R	R	R	R	R	R	R	R	R	R	R	R
**ATM**	R	R	R	R	S	R	R	R	R	R	R	S	R	R	R	S	R	R	R	R	R	R
**DO**	S	S	S	S	R	S	S	S	S	S	S	S	S	S	S	S	S	S	S	S	S	S
**TE**	S	R	R	S	R	S	R	S	S	S	S	R	S	R	R	S	R	R	S	S	S	R
**CIP**	R	R	S	R	S	R	R	R	R	R	R	R	R	R	R	R	R	S	R	R	S	R
**LEV**	R	R	S	R	R	S	R	S	R	R	R	S	S	R	R	R	R	S	R	R	S	S
**AK**	R	R	R	S	R	S	R	S	S	R	S	R	R	S	S	S	R	R	R	S	S	S
**CN**	R	R	R	S	R	S	R	S	S	R	R	R	S	S	R	S	R	S	R	S	S	R
**TOB**	R	R	R	R	R	S	R	R	R	R	R	R	S	S	R	R	R	R	R	R	R	R
**SXT**	R	S	R	R	R	R	R	R	R	S	S	R	R	R	R	S	R	R	S	S	S	R
**AZM**	R	R	R	R	R	S	R	S	R	R	R	R	S	R	S	R	S	R	R	R	S	R
**DDST**	+	-	+	+	+	-	+	-	+	+	+	+	-	+	+	+	+	+	-	+	+	-
**MHT**	+	+	+	-	+	-	+	+	+	+	+	+	-	+	+	+	+	+	+	+	-	+
***bla*VIM**	-	-	-	-	+	-	-	-	-	+	-	+	-	-	-	-	+	-	-	-	-	-
***bla*NDM**	+	-	+	-	+	+	-	-	+	+	-	+	-	+	+	+	-	+	+	+	-	+
***bla*OXA**	-	+	+	-	-	-	+	+	+	-	+	-	-	+	-	+	+	-	-	+	-	+
***bla*CTX-M**	+	+	+	+	-	+	+	-	+	+	+	-	-	+	+	+	+	+	-	+	-	-
***bla*SHV**	+	+	+	-	-	-	+	-	-	+	-	+	-	-	+	+	+	+	-	-	-	-
***bla*TEM**	-	+	+	-	-	-	+	-	-	+	+	+	-	-	+	-	+	-	+	+	-	-
aac(6')-Ib	+	+	+	+	+	-	+	+	+	+	+	+	+	+	+	+	+	+	+	+	-	+
acrA	+	+	+	+	+	+	+	-	+	+	+	+	+	-	+	+	+	+	-	+	+	+
**MEM MIC (μg/ml)**	**64**	**32**	**512**	**16**	**64**	**32**	**256**	**256**	**512**	**>512**	**64**	**32**	**8**	**>512**	**32**	**64**	**64**	**>512**	**32**	**>512**	**64**	**>512**

Abbreviations: AA: antimicrobial agent, S: sensitive, I: intermediate, R: resistant, AX: amoxicillin, AMC: co-amoxiclav, CFR: cefadroxil, CXM: cefuroxime, CTX: cefotaxime, FEP: cefepime, MEM: meropenem, ATM: aztreonam, DO: doxycycline, TE: tetracycline, CIP: ciprofloxacin, LEV: levofloxacin, AK: amikacin, CN: gentamicin, TOB: tobramycin, SXT: co-trimoxazole, AZM: azithromycin, DDST: double disk synergy test, MHT: modified Hodge test.

Table S4. Carbapenem-resistant *P. aeruginosa* isolates

AA	P2	P4	P20	P22	P23	P30	P63	P67	P109	P110	P115	P143
**AX**	R	R	R	R	R	R	R	R	R	R	R	R
**AMC**	R	R	R	R	R	R	R	R	R	R	R	R
**CFR**	R	R	R	R	R	R	R	R	R	R	R	R
**CXM**	R	R	R	R	R	R	R	R	R	R	R	R
**CTX**	R	R	R	R	R	R	R	R	R	R	R	R
**FEP**	R	R	R	R	R	R	R	R	R	R	R	R
**MEM**	R	R	R	R	R	R	R	R	R	R	R	R
**ATM**	S	R	S	S	S	R	R	R	S	S	R	S
**DO**	R	R	R	R	R	R	R	S	S	S	S	S
**TE**	R	R	R	R	R	R	R	S	S	R	S	S
**CIP**	R	R	R	R	R	R	R	R	R	R	R	R
**LEV**	R	R	R	R	R	R	R	R	R	R	R	R
**AK**	R	S	R	R	R	R	S	R	S	S	R	R
**CN**	R	R	R	R	R	R	R	S	S	S	R	S
**TOB**	R	R	R	R	R	R	R	R	R	S	R	R
**SXT**	R	R	R	R	R	R	R	R	R	R	S	R
**DDST**	-	-	-	-	-	-	-	+	-	+	+	-
**MHT**	-	+	-	-	-	-	-	+	-	-	+	-
***bla*VIM**	-	-	-	-	-	-	-	-	+	+	-	+
***bla*NDM**	-	-	-	-	-	-	-	+	-	-	-	-
***bla*OXA**	-	+	-	-	-	-	-	+	-	-	+	-
***bla*CTX-M**	+	+	+	+	+	+	+	+	-	-	-	-
***bla*SHV**	+	+	+	+	+	+	+	+	+	-	+	+
***bla*TEM**	+	+	+	-	+	+	+	-	-	-	+	-
aac(6′)-Ib	+	+	+	+	+	+	+	+	-	-	+	+
mexA	+	+	+	+	+	+	+	+	+	+	+	+
**MEM MIC (μg/ml)**	64	32	16	32	256	64	32	>512	>512	>512	>512	>512

Abbreviations: AA: antimicrobial agent, S: sensitive, I: intermediate, R: resistant, AX: amoxicillin, AMC: co-amoxiclav, CFR: cefadroxil, CXM: cefuroxime, CTX: cefotaxime, FEP: cefepime, MEM: meropenem, ATM: aztreonam, DO: doxycycline, TE: tetracycline, CIP: ciprofloxacin, LEV: levofloxacin, AK: amikacin, CN: gentamicin, TOB: tobramycin, SXT: co-trimoxazole, DDST: double disk synergy test, MHT: modified Hodge test.

Table S5. The NCBI accession codes of the genes detected in this study

Genes	Encoded Proteins	Isolates	Accession Codes
***bla*$_{NDM}$**	Class B carbapenemase NDM	*K. pneumoniae K62*	MH971065
***bla*$_{NDM}$**	Class B carbapenemase NDM	*K. pneumoniae K76*	MK341121
***bla*$_{VIM}$**	Class B carbapenemase VIM	*P. aeruginosa P143*	MW219607
***bla*$_{OXA-48}$**	Class D carbapenemase OXA-48	*K. pneumoniae K83*	MK341123
***bla*$_{OXA-48}$**	Class D carbapenemase OXA-48	*K. pneumoniae K43*	MK468795
***bla*$_{CTX-M}$**	Class A ESBL CTX-M	*K. pneumoniae K38*	MK341126
***bla*$_{SHV}$**	Class A ESBL SHV	*K. pneumoniae K44*	MK482385
***bla*$_{TEM}$**	Class A ESBL TEM	*K. pneumoniae K38*	MK341127
aac(6′)-Ib-cr	Aminoglycoside acetyl transferase (6′)-Ib	*K. pneumoniae K1*	MK493336
mexA	RND efflux pump MexA subunit	*P. aeruginosa P20*	MK341124
acrA	RND efflux pump AcrA subunit	*K. pneumoniae K76*	MK468796
acrA	RND efflux pump AcrA subunit	*K. pneumoniae K6*	MK468797
